# Supplementary material for: A Humanized Yeast Phenomic Model of Deoxycytidine Kinase to Predict Genetic Buffering of Nucleoside Analog Cytotoxicity
Source: Genes (Basel). 2019 Sep 30;10(10):770. doi: 10.3390/genes10100770 (PMC6826991; doi:10.3390/genes10100770)
Supplement: Supplementary file 1 [file genes-10-00770-s001.zip › Additional_Files/Additional_File7_GOTermHeatmaps.zip!/Additional_File7_GOTermHeatmaps/Swr1_complex.pdf]

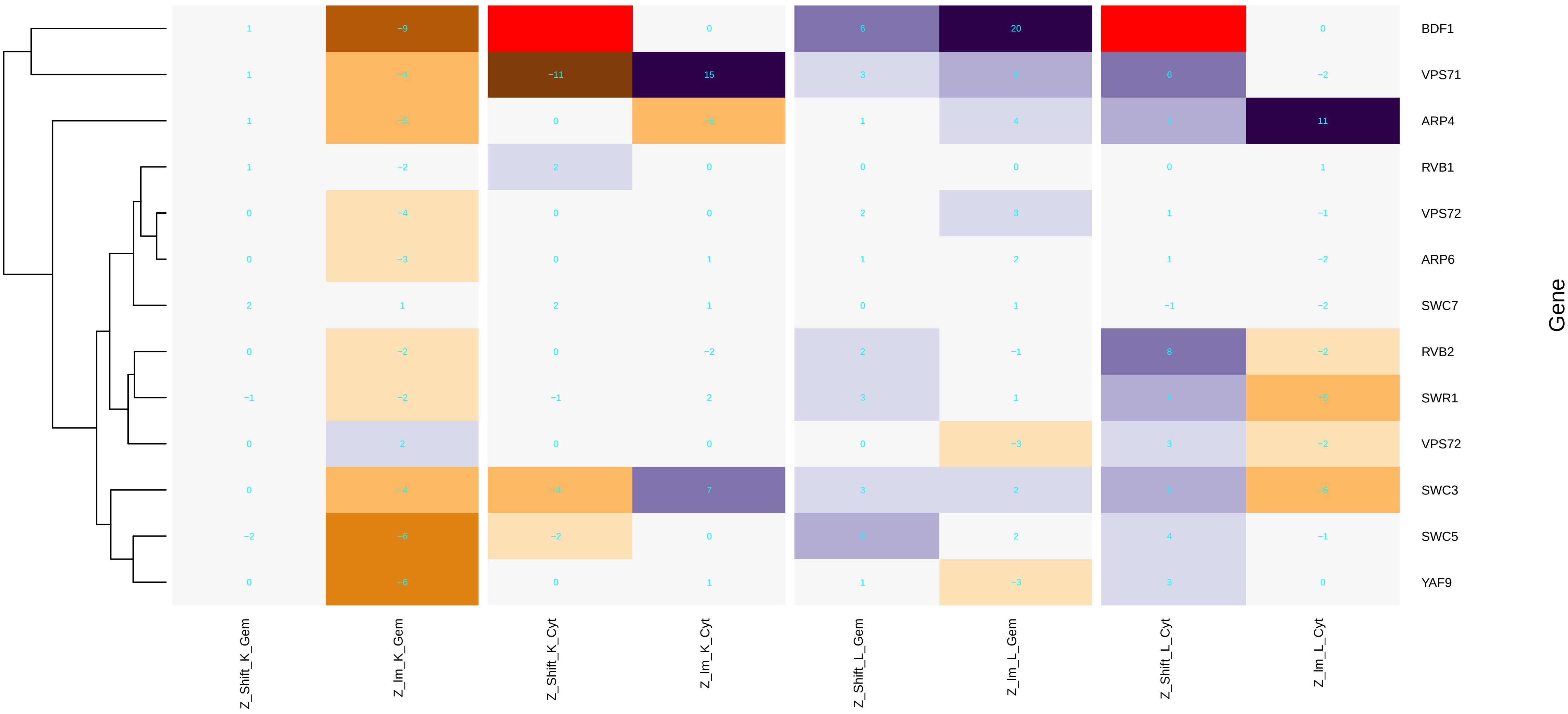

Z_Shift_K_Gem
Z_Im_K_Gem
Z_Shift_K_Cyt
Z_Im_K_Cyt
Z_Shift_L_Gem
Z_Im_L_Gem
Z_Shift_L_Cyt
Z_Im_L_Cyt
Gene
BDF1 1 −9 0 6 20 0
VPS71 1 −4 −11 15 3 5 6 −2
ARP4 1 −5 0 −6 1 4 4 11
RVB1 1 −2 2 0 0 0 0 1
VPS72 0 −4 0 0 2 3 1 −1
ARP6 0 −3 0 1 1 2 1 −2
SWC7 2 1 2 1 0 1 −1 −2
RVB2 0 −2 0 −2 2 −1 8 −2
SWR1 −1 −2 −1 2 3 1 4 −5
VPS72 0 2 0 0 0 −3 3 −2
SWC3 0 −4 −4 7 3 2 5 −6
SWC5 −2 −6 −2 0 5 2 4 −1
YAF9 0 −6 0 1 1 −3 3 0
